
JOURNAL INFORMATION
==============================
NLM Title Abbreviation: Atlanta Med Surg J (1884)
NLM Journal ID: 101251623

Atlanta Medical and Surgical Journal (1884)

Publisher: Biomedical Journal Digitization Project

ARTICLE INFORMATION
==============================
PMCID: PMC9201841
PMID: 35829598
Article version: 1
Subjects: Editorial

Hygiene in Shaving and Hair Dressing Parlors

Print publication date: 1899 Jan
Volume: 15
Volume Series: New Ser
Issue: 11
First page: 749
Last page: 756
License: This work is free of known copyright restrictions. For more information, please see PMC Back Issue Digitization.
License URL: https://creativecommons.org/publicdomain/mark/1.0/

==============================

a most important function, in procuring, each in his own State, that support on the floor of Congress which is essential to the satisfactory maintenance of a public department, a support which cannot easily be refused to one who can speak with authority among the constituents of the respective members of Congress."

### HYGIENE IN SHAVING AND HAIR-DRESSING PARLORS.

It has always been remarkable to us that more skin diseases are not contracted in barber-shops or at least can be traced to this source, and yet if the truth were known we believe that there are even more than are known. The unhygienic manner in which the large majority of these shops are managed would certainly seem to make them "contagion shops" for various skin diseases. For the good of the public we believe that men should be educated to see the error of being attended in barber-shops by men who themselves look uncleanly and use instruments which pass over a hundred faces in one day without even being cleaned. If these shops are to be patronized, and there is no doubt but that they always will be, then such frequenters should have their own razor, and brush and soap. We believe that every man who shaves should learn to shave himself, for it is in that case always done better and is far more cleanly. In an editorial, the *Canadian Medical Record* reviews the action of the board of health of the province of Quebec, and the views there expressed are certainly worthy of consideration. It says:

"The board of health of the province of Quebec has issued a circular, and caused it to be published in the newspapers (a copy of which we give below), which in a commendable manner aims at mitigating a well-recognized source of danger in regard to the spread of infectious diseases. The dangers to which persons are exposed in the manner indicated by the circular are not appreciated by people generally, and a single publication in the daily papers is hardly sufficient to educate the ordinary mind to the real danger incurred by their barber using the same instruments promiscuously without proper cleansing. It will only be when the public demand the changes indicated, that barbers will take the necessary precautions and carry out the directions so scientifically tabulated in the circular. A properly educated public will go farther than the barber-shop. They will inquire whether the restaurant-keepers disinfect the eating utensils which are used several times daily by different people. Whether dentists render aseptic the instruments which enter a dozen or more mouths each day. Danger on these lines lurks on all sides; even the sacred book in our court-houses may be the medium of conveying disease from one mouth to another, and the parched wayfarer who may quench his thirst at a public fountain may, unconsciously, with the refreshing waters imbibe specific contagion from the drinking-cup. We heartily indorse the action of the board in sounding this warning note, and hope they will not cease their agitations with the present efforts, but will continue to rouse the community in regard to the great dangers that insidiously lurk and claim victims who are unconscious of the existing perils.

"Beyond all doubt, any one who patronizes a barber or hairdresser, whose establishment is open to all comers, runs great risk of becoming infected with disease, from the razor, shaving-brush, scissors, clipper, comb or hair-brush having been previously used on a sick person or even on a corpse.

"That the chance of contagion is much less when the establishment is well kept, we most willingly admit; that the danger of infection is minimized in first-class establishments, we also concede, but the truth, nevertheless, obliges us to declare this:

"At the present moment, in the whole province of Quebec, there is not a single barber or hairdresser who can honestly say that his instruments are absolutely, completely and scientifically safe in regard to the possibility of conveying infection.

"Should a barber or hairdresser, recognizing the danger of transmitting, by the use of his instruments, certain infectious or parasitic diseases, amongst which may be mentioned the most terrible, and at the same time, perhaps, most frequently met with disease, syphilis, be willing to take the necessary antiseptic measures and to offer to his customers a guarantee of the most minute cleanliness conscientiously carried out, he may rest assured of an immense increase of his clientele, as the people of this province are now thoroughly aroused to the necessity of taking preventive measures against contagious disease in all forms.

"What then should be done to put hair-dressing parlors in a desirable hygenic condition?

"The board of health of the province of Quebec, which has the supervision and care of the public health in this province, thinks it opportune to make public the conclusions of a report, duly approved by it, which prescribes the best means of avoiding the dangers which necessarily arise from the use in common of the razor, shaving-brush, scissors, clipper, comb and hair-brush."

---

IN the December 3d issue of the *Philadelphia Medical Journal* there appears the following notice which will explain itself:

"For reasons set forth in another column, this *Journal* will, on and after December 31st, discontinue all exchanges with other journals. Publishers and mailing clerks will kindly bear this in mind, and from the date mentioned cease to mail us their journals on account of exchange. The business manager of the *Journal* will soon remit the regular subscription price for the journals desired by us for 1899."

This action of the *Philadelphia Medical Journal* is indeed a new departure in journalism and yet those who have watched the peculiar idiosyncrasies of the general get-up of this journal could not be surprised at this suddenly manifested eccentricity. The motives of the management of this journal may certainly be the best in the world, but we do not think that it has shown good judgment in this one act. In extenuation of this peculiar change, the editorial management says: "If the exchange courtesy is extended to a score, it is asked by hundreds of others, until each finds that he is spending a large amount of money, perhaps a thousand dollars annually, in sending out his own copies and for which he receives several hundred journals, the greater number of which, in his special field of work, are worthless." This is the apology. We have yet to see the time when we cannot choose our own friends, and we had sooner select those journals with which we wished to exchange than to show discourtesy to all by isolating ourselves to "single blessedness." It is true that there are a great many exchanges received which are worthless and could show no just cause for existence, but journals as individuals certainly have the right to select their own friends. Of course every journal which is backed by ready cash can afford to publish its pages for the delectation of its own editorial staff, but we think that the broad principles of professional courtesy should be an attribute of every medical periodical, even though its height of perfection could never be attained by others.

---

AT the recent Quarantine Convention held in Memphis, composed of representatives from all the Southern States, the following resolution was unanimously adopted as expressing the views of those present:

"The committee on resolutions, after careful consideration of the many valuable resolutions offered by the various members of the convention, have decided upon the following as embodying the ideas expressed in the majority of the said resolutions:

"Therefore, That for the purpose of protecting and improving the general health of the people of the United States, co-ordinating and harmonizing the action of the State and National sanitary authorities; framing regulations for the treatment of infected vessels and material at all infected or suspected foreign ports of shipment; preventing unnecessary interference with commerce, the United States mail, or through traffic by land or water, and for adopting a uniform system of quarantine for all ports in this country; be it

"*Resolved*, That there be established on a broad and comprehensive basis a National Bureau of Public Health in the Department of the Treasury of the United States, that the administration of *all* the public health functions now exercised by authority of the United States be placed in the hands of this Bureau.

"2. That the sanitary authorities and commercial interests of the several States of the Union be brought into immediate relations with the Bureau and be given a due share in the power and responsibilities of the central board through the agency of an advisory council consisting of one member from each State, to be appointed by the authorities of the several States."

---

SPEAKING of the "absorption treatment" of cataract reminds us of the suit for damages recently instituted by a patient who had lost both eyes while an inmate of the celebrated "Bemis Sanatorium." This "Bemis Sanatorium" is advertised most especially in the weekly and monthly popular periodicals, and appeals to the laity for support by holding out to them most marvelous cures for every eye disease without the use of the knife. Such an advertisement touches a popular chord, because most people have a dread of the word "operation," and like the majority of the negro race, they had rather lose the whole eye than to submit to an operation of painless character. Even as far south as Atlanta, we have run across patients with fully ripened cataracts who are wholly content with the use of the "eye vapor" furnished from the above institution, and are fully convinced of its curative effect, with all the child-like faith of a Christian scientist. Recently a patient who was at the sanatorium to have his cataract absorbed, developed gonorrheal ophthalmia, and as a result lost his eye. It was found that there was a case of this ophthalmia which had come for treatment, and the physician in charge had used the same medicine dropper on the other patients, and had thus conveyed the contagious material. This shows the scientific character of the institution! When the patient consulted a physician outside of the institution and found out the real condition of his eyes there was, of course, "blood on the moon." As a result of this, we believe he has instituted a suit for damages to the amount of $20,000, but so far the result of it has not been learned.

---

THE heralding of new remedies as a cure-all for eye diseases, and as for that matter, for every portion of the human anatomy, occurs now almost daily. We have received pamphlets from time to time from the Walker Pharmacal Company, extolling a new remedy called by them "Cineraria Martima," for the cure of cataract by gradual absorption. The "absorption treatment" of cataract is an old fake, and every now and then crops out in a new guise. Recently Dr. J. Ellis Jennings, of St. Louis, decided to consult the Walker Company, and with their aid and permission to give the remedy a thorough test. He found this pharmacal company located above a Homeopathic pharmacy, and in charge of a Homeopathic physician. The doctor stated his mission and asked to be given some new facts concerning this wonderful remedy. He stated to the gentleman in charge that such reports as had been sent out, usually mere statements of the patient, could not be relied upon and were of no value. As the circular stated that "leading physicians and ophthalmologists were using the prepration with generally good success," he asked to see a report from an ophthalmologist. They had none. He then asked if they would be willing to furnish him with a quantity of the drug so that he could test its value in a number of selected cases. After consultation they refused to grant his request. In conclusion Dr. Jennings states, "that so far as I could learn there is no evidence that Cineraria Martima is of any value as a curative agent in cataract, and from the action of the Walker Chemical Co., they evidently are not anxious to have the drug properly tested."

---

DR. FRANK S. BOURNS, who was commissioned on General Merritt's staff, and sailed on the first transports for Manila, in the Philippine Islands during the summer, is now Major and President of the Board of Health of that Philippine city. Dr. Bourns's former residence in the Philippine Islands and his ability to speak the language of these islands, in addition to his marked executive ability both as a physician and as an army man, has made his services invaluable to the commanding General, and hence we are not surprised to hear that the doctor has been placed in entire charge of the Medical Department of Manila and is the President of the Board of Health. He writes, however, that he is very anxious to return to Atlanta to resume his old work, as he is heartily tired even of his exalted position in the Pacific islands. Now that the United States will come into the possession of these islands there may be yet higher honors in store for Dr. Bourns, and Atlanta may yet lose him as a citizen.

WE wish to call attention to the splendid offer that this JOURNAL makes to its subscribers for the coming year. We will send to each cash subscriber this JOURNAL and the *International Journal of Surgery* for the one subscription price of two dollars. This is a splendid offer for any physician who wishes to read the best literature on medicine and surgery. In the coming year we expect to make this JOURNAL more interesting than ever, not only by its original articles, but by articles taken from other journals in every department of medicine. This is the official organ of the State Medical Examining Board, and will give the questions and the list of successful candidates after each examination. We expect to report the proceedings of the Atlanta Society of Medicine and to give medical items from all over the State. We ask all of our friends to send us items of interest and we will cheerfully publish them.

---

THE following officers were elected in the Atlanta Society of Medicine for the year 1899:

President—Dr. W. S. Goldsmith.

Vice-President—Dr. Katherine Collins.

Secretary—Dr. Claude A. Smith.

Treasurer—Dr. E. Van Goidtsnoven.

Librarian—Dr. Mike Hoke.

The Society has also instituted a movement for the thorough organization of a medical library, and in the next issue we will speak more at length in regard to this.

---

**NOTICE.—This JOURNAL and THE INTERNATIONAL JOURNAL OF SURGERY one year for $2.00 cash.**
